# Smart Buildings IoT Networks Accuracy Evolution Prediction to Improve Their Reliability Using a Lotka–Volterra Ecosystem Model

**DOI:** 10.3390/s19214642

**Published:** 2019-10-25

**Authors:** Roberto Casado-Vara, Angel Canal-Alonso, Angel Martin-del Rey, Fernando De la Prieta, Javier Prieto

**Affiliations:** 1 BISITE Research Group, University of Salamanca, Edificio Multiusos I+D+i, 37007 Salamanca, Spain; acanal@usal.es (A.C.-A.); fer@usal.es (F.D.l.P.); javierp@usal.es (J.P.); 2 Department of Applied Mathematics, Institute of Fundamental Physics and Mathematics, University of Salamanca, Calle del Parque 2, 37008 Salamanca, Spain; delrey@usal.es

**Keywords:** Internet of Things, Lotka–Volterra model, predator–prey system, bio-inspired system evolution, algorithm design

## Abstract

Internet of Things (IoT) is the paradigm that has largely contributed to the development of smart buildings in our society. This technology makes it possible to monitor all aspects of the smart building and to improve its operation. One of the main challenges encountered by IoT networks is that the the data they collect may be unreliable since IoT devices can lose accuracy for several reasons (sensor wear, sensor aging, poorly constructed buildings, etc.). The aim of our work is to study the evolution of IoT networks over time in smart buildings. The hypothesis we have tested is that, by amplifying the Lotka–Volterra equations as a community of living organisms (an ecosystem model), the reliability of the system and its components can be predicted. This model comprises a set of differential equations that describe the relationship between an IoT network and multiple IoT devices. Based on the Lotka–Volterra model, in this article, we propose a model in which the predators are the non-precision IoT devices and the prey are the precision IoT devices. Furthermore, a third species is introduced, the maintenance staff, which will impact the interaction between both species, helping the prey to survive within the ecosystem. This is the first Lotka–Volterra model that is applied in the field of IoT. Our work establishes a proof of concept in the field and opens a wide spectrum of applications for biology models to be applied in IoT.

## 1. Introduction

### 1.1. Problem Formulation

Internet of Things (IoT) networks are a current solution to a wide spectrum of technological problems. The range of applications varies from environment control and security to home automation and security control [1]. IoT networks relay the information provided by sensors in the IoT nodes. Data from the sensors are integrated and processed by the control algorithm that produces actuator signals. Nonetheless, faulty data due to inaccurate IoT nodes cause the algorithm to make erroneous decisions. Given that sensors are accurate (AD) in a normal state, we can define an inaccurate (NAD) state as an abnormal state caused by a malfunction, or even a False Data Injection attack [2]. The detection of this inaccurate state is often a complex task due to the volume of data and the protocols based on computing the average, sum, or minimum of the data [3]. Modelling the behavior of AD and NAD in IoT networks will allow us to understand its dynamics, but the complexity of these networks, caused by the large number of nodes, makes it impossible to use a conventional mathematical approach. Instead of calculating a failure rate in the NAD, we aim to model the IoT networks to accurately predict the behavior of the IoT nodes.

### 1.2. Motivation

The scientific community has studied this problem thoroughly, proposing several methods to cope with inaccurate sensors. The first solution against an inaccurate sensor is replacing it, but this implies detecting the error and identifying the faulty sensor, which is not always possible as seen in [4]. Another approach is designing control algorithms capable of coping with the input of a faulty sensor; this solution has been proposed in several published works [5,6] achieving good results. However, the scope of several of those researches was limited to the study of the NAD and AD in a discrete time, lacking a continuous approach. To achieve this, we propose the use of an innovative biological approach to solve this problem: the Lotka–Volterra (LV) predator–prey model [7]. The LV predator–prey model adjusts sensor populations better than other ecological models due to its simplicity and versatility. In order to study the reliability of IoT systems, there are several techniques and mathematical models such as probabilistic risk analysis using failure trees [8,9,10,11], Markov Chains [12,13,14,15], and Stochastic Petri Nets [16,17,18]. These models have been shown to perform well in predicting network confidence, future sensor accuracy states, etc. However, the aim of this article is to study the dynamics of accurate and inaccurate sensor populations in the IoT system in order to predict the population densities of each of the species and thus validate the confidence of the IoT network by studying the interaction between the species that make up that ecosystem (IoT network). For these reasons, we chose the LV model to make a theoretical approximation of this problem in our research.

In this paper, an IoT network is represented as a predator–prey system by means of a state space model in which the predators are the inaccurate sensors and the prey are the accurate sensors. The model identifies the population density of each species. The dynamic modeling of the predator–prey systems makes it possible to quantitatively describe the interaction between populations, thus facilitating the definition and evaluation of ecosystem control strategies (which in this case is the IoT network). This technique makes it possible to predict the sensor population (both accurate and inaccurate sensors) and the level of damage to the ecosystem (IoT network), the efficiency, and the time the predators (non-precision sensors) need to dominate the prey (i.e., the IoT network has more sensors that are inaccurate than accurate). Furthermore, this study considers the intervention of a third species (maintenance technicians) who “capture” predators and return prey to the ecosystem (i.e., they take inaccurate sensors and fix or replace them, returning an accurate sensor to the network). Therefore, what we did was to model the Lotka–Volterra predator–prey system with the intervention of a third species, for the study of the dynamics of populations of accurate and inaccurate sensors in an IoT network.

### 1.3. Model Description

This novel approach assumes that sensors can be studied as a population and therefore that population dynamics models can be applied to them, although originally designed for biological entities. Despite the fact that IoT networks are not made of biological organism, their behavior resembles an ecosystem, which is composed of IoT nodes, sensors and actuators. Different types of sensors and actuators can be grouped as “species”: in this case, we will consider accurate sensors as one species and inaccurate sensors as another species. The species in an ecosystem interact between themselves (intraspecific interaction) and between other species (interspecific interaction).

The latter case perfectly describes the interaction between accurate sensors and inaccurate sensors. As said above, we propose the use of an LV model, originally proposed in 1910 as equations for periodic chemical reactions, focused on a non-competitive predator–prey model (since the populations do not compete with each other for the resources). The LV model makes the calculus of the population density and the growth rates of the two species possible. The LV model is based on first-order nonlinear differential equations that only take into account the rate of interactions between the prey species and the predator species. We have to set the following limitations in our model [19]:

1. Predator and prey populations are limited by the maximum number of sensors in the IoT network (i.e., inaccurate + accurate sensors = total amount of sensors in the IoT network.)
2. When there are no accurate sensors (prey) in the IoT network (i.e., all sensors are inaccurate), maintenance staff will be required to repair or replace inaccurate sensors.
3. The total number of prey that predators can consume will be less than or equal to the total number of sensors in the IoT network.
4. Both populations encounter each other randomly in the environment.

Notwithstanding, in a biological predator–prey relationship, the LV model has major limitations due to the last assumption; in an artificial ecosystem, such as that made up of IoT networks, the precision and reliability of the LV model is considerably increased. IoT sensors are not limited by resources, since they do not need anything to maintain their populations and therefore the first LV assumption applies to them. Moreover, NAD cannot reproduce without AD, also there is no limit (other than the total number of devices on how many devices can turn NAD, fulfilling the requirements for the second and third assumptions.

Consider this case with only two species, which we will call predator and prey, that coexist in a common ecosystem. It is represented by x(t) and y(t) the number of individuals of the prey and predator species respectively. The equations initially proposed by Volterra can be expressed as follows:

(1) $$\begin{array}{cc} \frac{dx}{dt} & =x(a-by),\\ \frac{dy}{dt} & =y(-c+dx), \end{array}$$

where a,b,c,d are positive constants. The meaning of the model is summarized as follows:

- In the absence of predators, y=0, the equation for the prey is reduced to $\frac{dx}{dt}=\alpha x$, being the intrinsic growth constant for *x*, until it reaches the total device number.
- In the absence of prey, x=0, the equation for the predator takes the form of $\frac{dy}{dt}=-cy$, which we know results in an exponential decrease and subsequent extinction (collapse) of the population (in this case, due to malfunctioning nodes substitution). As a consequence, *c* is therefore the intrinsic rate of decrease of *y*.
- The constant b>0, which corresponds to the term crossed -bxy in the first equation, shows that the interactions between the two species, which are assumed to be proportional to the xy product of both populations, are unfavorable for the prey (hence the negative sign).
- Similarly, the constant d>0 corresponds to the crossed term dxy in the second equation, which shows that encounters between individuals of both species are favorable for the predator.

### 1.4. Main Results

This paper addresses the research gaps in the monitoring of continuous time networked systems with multiple IoT devices, with the aim of presenting a model that will help achieve the maximum possible efficiency in predictive maintenance. A unified bio-inspired model is presented in continuous time with one to improve the prediction of the precision states of IoT devices. The output of the algorithm is the continuous time prediction of the operating state of the IoT network (i.e., behavior of the IoT network). The main contribution of this document can be summarized as follows:

1. As far as we know, this is the first time the behavior of AD and NAD is studied as an ecological system using predator–prey population models, being a ground-breaking approach that opens up many opportunities for new research as it is the first time an LV model has been applied to IoT study. This also has direct economic interest, as it has the potential to be applied in preventing economic loss caused by NAD.
2. A novel approach for estimating the operating states of IoT nodes from a bio-inspired model of predator–prey relationships. The behavior of accurate and inaccurate sensors in the IoT network is also studied, knowledge of which will allow to optimise its operation of the IoT network

The efficiency of the presented approach is illustrated by two selected simulations.

This paper is organized as follows: Section 1 overviews works related to the key aspects of this article. The proposal is presented in detail in Section 2. Section 3 presents two simulations, the results of which validate the competency of the proposal. Finally, Section 4 draws conclusions from the conducted study and describes future lines of research.

## 2. Proposed Approach

In this paper, we will address the study of dynamical systems that describes the interaction between two species that coexist in the same common ecosystem. This model is closer to those of a single species, found in real situations in biology and ecology.

### 2.1. Successful Applications of the LV Model in Other Fields

In this paper, we propose a new model that studies the behavior of an IoT network with the predator–prey approach with external intromission of a third species (smart building maintenance staff). Although the LV approach is often used in animal ecosystems, there are some successful cases of the application of the LV model to other fields such as economy [20,21,22], chemistry [23,24,25], and infectious diseases [26,27] to name but a few. We have conducted an in-depth study of some of the most transferable applications in the industry, demonstrating that our approach is novel in the field of IoT and cannot be compared to other similar techniques. In [28], the authors explore the growth of Taiwan’s 200 mm and 300 mm silicon wafers. The authors also analyze the dynamic growth of the competitive relationship between 200 mm silicon wafers and 300 mm silicon wafers. They also perform equilibrium analysis to determine the long-term stability state in the simulation trajectory. This investigation shows that 200 mm silicon wafers and 300 mm silicon wafers show a predator–prey relationship under the assumption of natural competition in the global semiconductor market. Gatabazi et al. propose the Lotka–Volterra Gray Fractional Model that is introduced and used to model the transaction counts of three cryptocurrencies (Bitcoin, Litecoin, and Ripple). The authors do a two-dimensional study on Bitcoin and Litecoin, while the three-dimensional study is on Bitcoin, Litecoin, and Ripple. The analyses made by the authors evoke a constant trend in the Bitcoin transaction and a decreasing trend in the Litecoin and Ripple transaction. Bitcoin will maintain relatively higher transaction counts, with Litecoin transaction counts higher than Ripple at all times [29,30]. Prakash et al. propose a non-autonomous predator–prey system with feedback controls. Some of its possible applications in some branches of advanced computing have also been discussed. The authors present some possible applications of the work in bioinformatics, social networks, etc. Having carried out an in-depth analysis of these papers, we found a gap in the applications of the Lotka–Volterra model in the field of IoT. We have found that the applications of the LV model in different fields are novel as is our proposal. However, based on the efficiency of the LV model in other fields, in this article, we present the application of the LV model in IoT.

### 2.2. LV System Applied to IoT Networks

**Figure 1:**
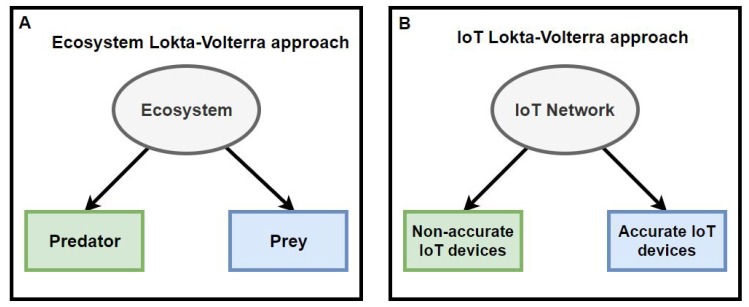
Modeling the Lotka–Volterra equations for an IoT network in a smart building. The classical approach of the Lotka Voterra system for the analysis of predator and prey dynamics in a biological ecosystem is presented in (**A**). Whereas in our approach (**B**), based on the biological approach of predator-prey dynamics, we have proposed a new model Lotka Volterra, in our approach the ecosystem is the IoT Network, the predators are the non-accurate devices and the prey are the accurate devices.

An IoT network can be considered as an ecosystem. An ecosystem is composed of multiple species that live together and interact with each other and with the non-living parts around them (i.e., resources) to find what is necessary to survive. Similarly, an IoT network has a large number of cooperative devices. These devices can have two states: accurate or inaccurate. If we consider these two states of the IoT devices as the two species surviving in the ecosystem (i.e., IoT network), studies of the evolution of populations in ecological systems for these two species can be applied. In addition, an important point is that we are going to take into account the temporal evolution of the populations of accurate devices and inaccurate devices. This assumes that the LV system will be non-autonomous, as discussed in Section 2.3. Our proposed approach can be found in Figure 1.

**Figure 2:**
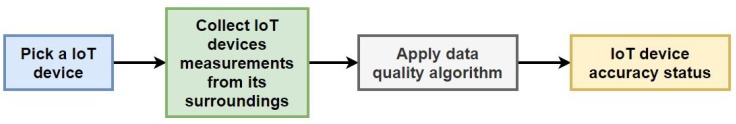
Flowchart to show how an IoT device is determined to be an accurate or inaccurate device.

Next, we are going to describe the necessary concepts to apply the LV equations in our proposed IoT network:

- Ecosystem:The ecosystem we are going to use for this model is an IoT network in a smart building. We have assumed that there will be two species in this ecosystem: (1) the accurate IoT devices. These devices collect data accurately or with an error below 15%, following Casado-Vara et al. in [6]. (2) The inaccurate IoT devices. These devices do not collect data accurately, with an error above 15%. In this ecosystem, we are going to consider that there is external interference, that is, a predator (inaccurate IoT device) can die through the external interference of maintenance personnel (removing it from the IoT network or repairing it). On the other hand, when maintenance personnel fix an IoT device or deploy a new one in the IoT network, we will consider it as the birth of a new prey in the ecosystem.
- Life cycle: The life cycle of IoT devices begins with their birth (i.e., the IoT devices are installed on the IoT network) and ends when they die (i.e., the IoT devices are removed from the IoT network). During its life cycle, an IoT device can be cured (repaired), which in our model would transform the predator (inaccurate IoT device) into a prey (accurate device).
- Predator: A species that lives in the ecosystem we are going to study (i.e., IoT network). We consider the inaccurate IoT devices as the predators.
- Prey: The accurate devices will be considered the prey in this ecosystem.
- Predator–prey interaction: The aim is to detail the predator–prey interaction. It is necessary to have this definition in mind since it is one of the key elements of predator–prey ecological models. In our ecosystem, we are going to consider that a predator kills a prey when a NAD gets an AD to start collecting erroneous data. In order to achieve this, the data quality algorithm developed by Casado-Vara et al. in [6] will be used. This algorithm makes a consensus between the IoT devices that are in the same area (i.e., they are the neighbors of the sensor whose precision is being observed); thus, applying this algorithm based on game theory, they can identify the IoT devices that are being accurate and inaccurate. In Figure 2, one can find a flowchart to shed some light to this “predator kills a prey” process.

This happens when several NADs in an IoT network surround a neighboring AD, the smart building control algorithm begins to consider the data collected by AD as erroneous and that of NADs as correct. This way, the AD will start collecting erroneous data and become an NAD. In other words, it is this modelling that we propose; predators transform prey into new predators (i.e., one could consider that our model is a zombie model). Meanwhile, human intervention in the ecosystem transforms predators into new prey.

**Figure 3:**
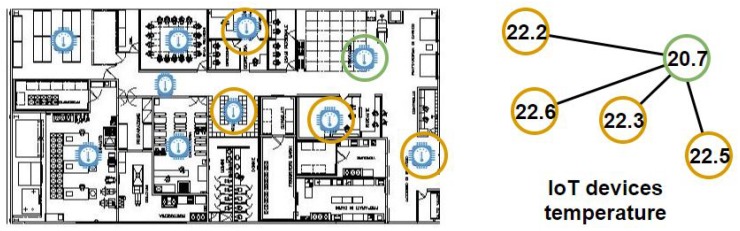
Smart building with an IoT network. The measurements from each device are sent to a consensus algorithm to be processed. There are five IoT temperature measurements, four from accurate IoT devices (orange), and another one from an inaccurate IoT device.

**Example** **1.** *In Figure 3, the temperatures of five IoT devices are shown. Each device measures the temperature of their environment and the consensus algorithm developed in [6]. The output is that the green IoT device is inaccurate when the data quality algorithm is applied to the green IoT device and its neighbourhood. This is how you decide whether an IoT device is accurate or not. Once the data quality algorithm is finished, the orange IoT devices are accurate and the green ones are inaccurate; this is how predators (NAD) hunt prey (AD).*

### 2.3. Lotka–Volterra Non-Autonomous System

In this subsection, we are going to describe the Lotka–Volterra non-autonomous system (LVNA) model in which there is interference from external agents (i.e., the maintenance staff fix or remove inaccurate IoT devices, and also deploy new IoT devices in the IoT network). The study of non-autonomous systems is important because the function depends on time; similarly, the accuracy of IoT devices is affected by time (i.e., IoT devices can lose accuracy over time). The novel contribution of this paper is that we add a term of auto-inhibition (intra-species competition) in order to prevent the explosion of predator populations (i.e., NAD) considering that the maximum NAD can be reached by the maximum number of IoT devices in the IoT network. When this happens, we will say that the IoT network is not reliable and, therefore, the use of different IoT devices will be recommended for the IoT network of that smart building. Therefore, doing a numerical simulation solving the LVNA equations can predict the behavior of the IoT network. It is possible to determine the degree of reliability of the IoT devices in terms of their useful life in the IoT network (e.g., it is possible to find out if the IoT devices’ work correctly for the period of time indicated by the manufacturer). In order to make these simulations, an LVNA model is used which arises from the study of blood coagulation in humans [31], in our study, we optimize this model to improve the precision of its results and adapt it to the study of the reliability of IoT devices in IoT networks. The LVNA system proposed in this paper is as follows:

(2) $$\begin{array}{cc} \frac{dx}{dt} & =\dot{x}=g\left(t\right)\;x\left(a-by\right),\\ \frac{dy}{dt} & =\dot{y}=y\left(-c+dx\right) \end{array}$$

for t>0, $\frac{dx}{dt}=\dot{x}$ is the derivative of x respect of time (Resp. for *y*) and the initial conditions are x(0)=y(0)=α. In this system, x(t) represents the population density of the predator (as its growth is favored by the presence of the prey) and y(t) is the population density of the prey, whose growth rate is reduced by the existence of predators. The g(t) function depends on time at x(t) such that g(t) will be a real and not negative function, whose value tends to zero when t⟶∞ (i.e., time *t* is large enough, in mathematical language this is expressed through the fact that t⟶∞) and should have the properties described in the next subsection.

#### 2.3.1. Properties of the Function g

We are now going to set the conditions that we want the g(t) function to fulfill; for this, we are going to rewrite Equation (2) as follows:

(3) $$\begin{array}{cc} \frac{dx}{dt} & =g_{\beta }\left(t\right)\;x\left(a-by\right),\\ \frac{dy}{dt} & =y\;(-c+dx), \end{array}$$

with β>0, the function class $g_{\beta }$ has to fulfill the following conditions:

- $g_{\beta }$ defined in [0,+∞) and takes values in (0,1] (i.e, $g_{\beta }:\left[0,+\infty \right)⟶\left(0,1\right]$).
- $g_{\beta }$ is continuous in [0,+∞).
- $g_{\beta }\left(0\right)=1$ and $g_{\beta }\left(t\right)<t$ for all t>0.
- $g_{\beta }\left(t\right)e^{\beta t}$ is upper and lower bounded when t⟶∞ (i.e., $g_{\beta }\left(t\right)e^{\beta t}\in \left[K_{0},K_{1}\right]$ with $K_{1}\geq K_{0}>0$ independent of *t*).

It is clear that the *g* function defined in Equation (2) fulfills the conditions required for $g_{\beta }$ just take β=1.

The novelty that we introduce in this paper is that we are going to study the long-term behavior of the solutions for Equation (3), which are the ones whose trajectories do not remain in a $\Omega_{j}$ for *t* big enough, nor converge to a point of the set *A*. In fact, it could happen that the trajectories will visit all the $\Omega_{j}$ infinite times. This happens in the case of $g_{\beta }=1$ where Equation (3) becomes a conventional LV system. In this way, we are going to model the behavior of human interference in the IoT network which occurs as a result of the need to repair the inaccurate IoT devices (the $g_{\beta }$ function models this behavior). Therefore, by mathematically modelling human behavior, all the data from the LV model are already available, and the main consequence is that the behavior of the IoT network can be studied to improve the maintenance of this IoT network in smart buildings. Some useful analytic results we will need regarding Equation (3) are demonstrated in Theorem 1. This theorem gives us a lot of information about the LVNA system solution we are proposing in this paper. This is important because we are modeling the behavior of IoT devices in an IoT network and the properties that are demonstrated in the theorem allow us to affirm that the results of the model will always be met. In this way, one could ensure that the result of the simulations will be very close to the real result, and this is supported by mathematically strong evidence (i.e., Theorem 1).

**Theorem** **1.** *Given the system described in Equation (3), let*

(4) $$\begin{array}{cc} \Omega_{1} & =\{(x,y):x>p,0<y<q\},\\ \Omega_{2} & =\{(x,y):0<x<q,p<y<q\},\\ \Omega_{3} & =\{(x,y):0<x<q,y>q\},\\ \Omega_{4} & =\{(x,y):x>p,y>q\} \end{array}$$

*be the partition of the first quadrant $C_{1}$. Then, the following properties are true:*

*1.*  *The interior of the first quadrant $C_{1}$, its border, and the point $\left(p,q\right)\in R^{2}$ are positively invariant sets of the system described in Equation (3).*
*2.*  *Let (x,y) be a solution of Equation (3) with initial conditions in $C_{1}$. If the solution is bounded and remains confined to a single $\Omega_{j}$ after a sufficiently long time, then it must converge to a point $\left(x_{\infty },y_{\infty }\right)$ belonging to the set:*
  (5) $$A=\left\{x=x_{0},v\geq 0\right\}\cup \left\{x\geq x_{0},y=0\right\}.$$
*If the solution remains confined to a $\Omega_{j}$, it cannot be $\Omega_{2}$ (trajectories in this region penetrate $\Omega_{3}$) or $\Omega_{4}$ (trajectories in this region penetrate $\Omega_{1}$).*
*3.*  *Let (x,y) be an Equation (3) solution that belongs to $\Omega_{3}$ for big enough t. Then, either (x,y) is delimited and converges to a point in {x=p,y=q} or $\left(x,y\right)⟶\left(x_{\infty },\infty \right)$ when t⟶∞ for some $x_{\infty }\in \left[p-\beta ,q\right]$.*

**Proof.**  See [31]. □

#### 2.3.2. Long-Term Behavior of Solutions

The purpose of this section is to prove that, if $g_{\beta }$ fulfills the required boundary condition when defining Equation (3), then the trajectory cannot visit the $\Omega_{j}$ infinite times, and the general situation is described by properties 2 and 3 of Theorem 1. In this way, if these conditions are met in the IoT network being monitored, it will be certain that the populations of inaccurate IoT devices will not experience a population explosion (i.e., the inaccurate IoT devices will always be a very small number within the IoT network). A possible population interpretation for the proposed LVNA system can be given in terms of the process of domestication of a wild species. Indeed, we can consider a population of wild cats (NAD in our model) that coexist in a given habitat with their preferred prey, field mice (AD in our model). In this situation, a predator–prey model from Lotka–Volterra describes the long-term behavior of cat and mouse populations in the first approximation. Suppose now that humans begin to artificially feed cats (e.g., fix NADs), so that they have progressively less need to hunt to ensure their survival. The term artificial feeding is a function of time $g_{\beta }\left(t\right)$ that is proportional to the growth rate of predators. This means that the growth of the cat population becomes independent of the hunting activity, and is therefore stabilized at a value that will depend on the carrying capacity (the number of cats that humans are willing to keep, i.e., the number of NADs they allow on the IoT network) but also on the initial conditions, as the system is no longer autonomous (i.e., no longer dependent on time). However, the hunting instinct of cats does not disappear completely because they are artificially fed, and they continue to hunt mice, even if they do not feed on them afterwards. This means that the equation for prey is not modified with respect to the situation prior to domestication. Applying this argument to our proposed model, this means that, even if NADs are repaired and transformed back into AD, the evolution of the system will tend to cause IoT devices to lose accuracy and become NADs again. Therefore, our approach to the solution is based on studying the behavior of IoT devices and making a study based on simulations and not on preventing IoT devices from becoming inaccurate.

**Theorem** **2.** *Let (x,y) be an arbitrary solution of Equation (3) in $C_{1}$. Then, (x,y) cannot visit any of the sets $\Omega_{j}$ infinite times and therefore cases 2 and 3 of Theorem 1 are the only possible solutions.*

**Proof.**  Introduce polar coordinates (r,θ) centered on (p,q) and take the clockwise direction as positive for θ. In addition, the θ angle is measured from the {(x,p):x>0}. Then, we have:

(6) $$\begin{array}{cc} x-p & =r\cos(\theta ),\\ y-q & =-r\sin(\theta ), \end{array}$$

so

(7) $$\begin{array}{cc} \dot{x} & =\dot{r}\cos\left(\theta \right)-r\left(\dot{\theta }\right)\sin\left(\theta \right),\\ \dot{y} & =-\dot{r}\sin\left(\theta \right)-r\left(\dot{\theta }\right)\cos\left(\theta \right), \end{array}$$

and, replacing in Equation (3), we get:

(8) $$\begin{array}{cc} g_{\beta }\;x\;\left(y-q\right) & =\dot{r}\cos\left(\theta \right)-r\left(\dot{\theta }\right)\sin\left(\theta \right),\\ y\;(p-x) & =-\dot{r}\sin\left(\theta \right)-r\left(\dot{\theta }\right)\cos\left(\theta \right). \end{array}$$

Now, multiplying the first equation by sin(θ) and the second by cos(θ), and adding both equations and simplifying we have:

(9) $$\dot{\theta }=g_{\beta }\;x\sin^{2}\left(\theta \right)+y\cos^{2}\left(\theta \right)=g_{\beta }\;x\;\left(1-\cos^{2}\left(\theta \right)\right)+y\;\cos^{2}\left(\theta \right),$$

so the equation for $\dot{\theta }$ is left:

(10) $$\dot{\theta }=g_{\beta }\left(t\right)\;x\left(t\right)+h\left(t\right)\;\cos^{2}\left(\theta \right).$$

Our goal is to prove that θ(t) is bounded in [0,∞]. Reasoning by reduction to the absurd: assume that the solution visits every $\Omega_{j}$ infinite times. Using Equation (10) given by a nonlinear version of the parameter variation method. We consider a non-constant solution of Equation (3) (x(t),y(t)) in $C_{1}$ defined for all t≥0. We will denote by $\theta (t,t_{0},\theta_{0})$ the solution of Equation (10) for $t_{0}\geq 0$ arbitrary with initial conditions $\theta \left(t_{0}\right)=\theta_{0}$. The only solution that makes sense in this case is $\theta (t,0,\theta_{0}),$ where $\theta_{0}\in \left[-\pi ,\pi \right]$ is the angle corresponding to (x(0),y(0)). For this demonstration, we are going to use the method of the variation of the constants in its nonlinear version (see [32,33,34,35]).

For each $t,t_{0}\geq 0$ and for each $\psi (t,t_{0},\psi_{0})$, the solution of the equation

(11) $$\dot{\psi }=h\left(t\right)\;\cos^{2}\left(\psi \right);\;\psi \left(t_{0}\right)=\psi_{0},$$

which is the homogeneous equivalent of Equation (10). Using as a script the demonstrations found in literature, we are going to follow an analogous procedure for the non-homogeneous equation of Equation (10). Indeed, Equation (11) admits an explicit solution of form:

(12) $$\tan\left(\psi \right)=\tan\left(\psi \right)+\int_{t_{0}}^{t}h\left(s\right)\;ds$$

to simplify notation $H\left(t,t_{0}\right)=\int_{t_{0}}^{t}h\left(s\right)\;ds$.

We define:

(13) $$\psi \left(t,t_{0},\psi_{0}\right)=\left\{\begin{array}{cc} arc\;\tan(\tan\left(\psi +H\left(t,t_{0}\right)\right),\; & if\;\psi_{0}\neq \frac{\pi }{2}+k\pi ,\;k\in Z,\\ -\frac{\pi }{2}, & if\;\psi_{0}=\frac{\pi }{2}+k\pi ,\;k\in Z, \end{array}\right.$$

and

(14) $$K\left(\psi_{0}\right)=\left[\frac{\psi_{0}}{\pi }+\frac{1}{2}\right]$$

Therefore, Equation (10) can be expressed as follows:

(15) $$\psi \left(t,t_{0},\psi_{0}\right)=K\left(\psi_{0}\right)\pi +\Psi \left(t,t_{0},\psi_{0}\right).$$

For the fundamental theory of differential equations, the application $\psi :{\left(R^{+}\right)}^{2}\times R⟶R$ is differential. Again, apply the constant variation method to find a function ϕ(t) such that $\phi \left(t_{0}\right)=\theta_{0}$ for all $t,t_{0}\geq 0$ and $\theta_{0}R$ with the equation

(16) $$\theta \left(t,t_{0},\theta_{0}\right)=\psi \left(t,t_{0},\phi \left(t\right)\right)$$

for all t≥0 in the maximum interval of existence of $\theta (t,t_{0},\theta_{0})$.

Thus, using the formula of the variation of the parameters:

(17) $$\dot{\theta }\left(t,t_{0},\theta_{0}\right)={\left.\frac{\partial \psi (t,t_{0},\psi_{0})}{\partial \psi_{0}}\right|}_{\psi_{0}=\psi \left(t\right)}\dot{\phi }\left(t\right)+h\left(t\right)\;cos^{2}\left(\psi \right),$$

we can conclude that ϕ(t) has to be a solution to the problem of initial values:

(18) $$\dot{\phi }\left(t\right)={\left[\frac{\partial \psi (t,t_{0},\psi_{0})}{\partial \psi_{0}}\right]}_{\psi_{0}=\psi \left(t\right)}^{-1}g_{\beta }\left(t\right)\;x\left(t\right),\;\phi \left(t_{0}\right)=\theta_{0}.$$

Doing the calculations in our case, you have:

(19) $$\begin{array}{cc} {\left[\frac{\partial \psi (t,t_{0},\psi_{0})}{\partial \psi_{0}}\right]}^{-1} & =\cos^{2}\left(\psi_{0}\right)\left[1+{\left(\tan\left(\psi_{0}\right)+H\left(t,t_{0}\right)\right)}^{2}\right]\\ \; & =\cos^{2}\left(\psi_{0}\right)+sin^{2}\left(\psi_{0}\right)+2\sin\left(\psi_{0}\right)\cos\left(\psi_{0}\right)H\left(t,t_{0}\right)\\ \; & +H^{2}\left(t,t_{0}\right)\cos^{2}\left(\psi \left(t\right)\right)\\ \; & =1+H\left(t,t_{0}\right)\sin\left(2\psi_{0}\right)+H^{2}\left(t,t_{0}\right)\cos^{2}\left(\phi \left(t\right)\right). \end{array}$$

Then,

(20) $${\left[\frac{\partial \psi (t,t_{0},\psi_{0})}{\partial \psi_{0}}\right]}_{\psi_{0}=\psi \left(t\right)}^{-1}=1+H\left(t,t_{0}\right)\sin\left(2\psi_{0}\right)+H^{2}\left(t,t_{0}\right)\cos^{2}\left(\phi \left(t\right)\right).$$

It is trivial to prove the fact that the solution (x,y) of Equation (3) visits every $\Omega_{j}$ infinite times implies that ϕ(t) is limited for t≥0, which, by the formula of the variation of the parameters we just obtained, implies the boundary of θ, which is an obvious contradiction.

For $t\geq t_{0}$, we have:

(21) $$\phi \left(t\right)\leq \theta_{0}+\int_{t_{0}}^{t}(1+|H\left(s,t_{0}\right)\left|+H^{2}\left(t,t_{0}\right)\right)\;g_{\beta }\left(s\right)\;x\left(s\right)\;ds.$$

Since $|h\left(t\right)|=|y\left(t\right)-g_{\beta }\left(t\right)\;x\left(t\right)|\leq y\left(t\right)-g_{\beta }\left(t\right)\;x\left(t\right)$ and, for the bound of $g_{\beta }$, we have $g_{\beta }\leq K_{1}e^{-\beta t}$, it is easy to prove that

(22) $$\begin{array}{cc} \int_{t_{0}}^{\infty }g_{\beta }\left(s\right)\;ds & <\infty ,\\ \int_{t_{0}}^{\infty }\left|H\left(s,t_{0}\right)\right|\;g_{\beta }\left(s\right)\;ds & <\infty ,\\ \int_{t_{0}}^{\infty }H^{2}\left(s,t_{0}\right)\;g_{\beta }\left(s\right)\;ds & <\infty . \end{array}$$

Then, ϕ(t) is bounded for $t\geq t_{0}$ and for t≥0. This implies that K(ϕ(t) is limited in t∈[0,+∞], which implies that $\phi (t,0,\phi_{0})$ is limited (arc tan is a bounded function). This implies that $\theta \left(t,t_{0},\theta_{0}\right)=\psi \left(t,0,\phi \left(t\right)\right)$ is bounded, which is a clear contradiction.

Therefore, for any non-constant solution (x,y) in $C_{1}$, the corresponding function of the polar angle θ is limited to t≥0, and such solution (x,y) cannot visit the $\Omega_{j}$ infinite times, which concludes the demonstration. □

## 3. Results

In this section, we will present two simulations to verify the efficiency of our new model. The objective of these simulations is to verify that the solutions described in the theorems of the previous section are true. For this purpose, two simulations are going to be designed with different parameters in which it is going to be possible to see, varying the growth rates of predator–prey and its efficiency, the behavior of the solutions for t≥0 should be sufficiently long.

### 3.1. General Description of the Experiment

**Figure 4:**
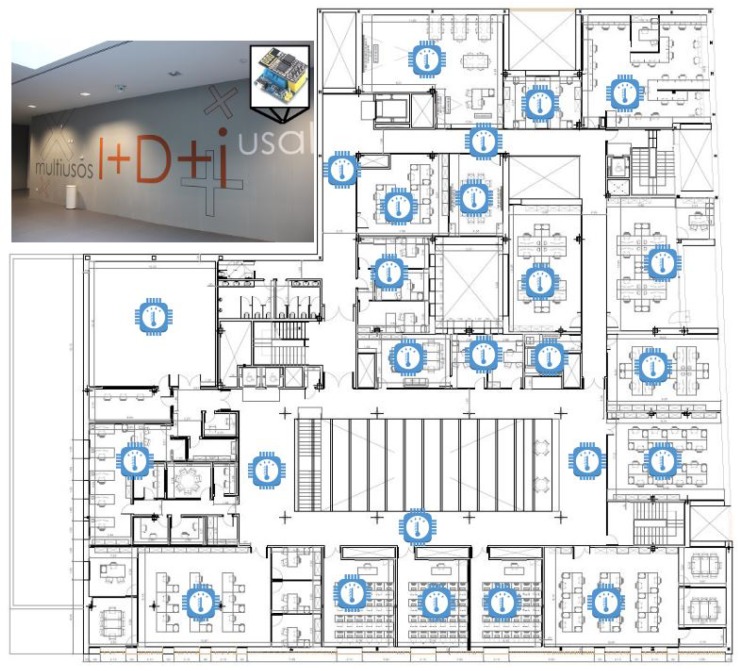
Map of the case study building. In this figure, IoT device location in the smart building is displayed.

To test the proposed model, we have chosen a smart building. The chosen smart building is the University of Salamanca’s R&D building, located on Espejo Street, a reference centre for the region of Castilla y Leon as well as nationally (Spain) and even internationally. Built between 2010 and 2014 with the latest materials and techniques for energy efficiency and an investment of 25 million euros (including equipment), the building has more than 13,000 square meters distributed over six floors, three of them in the basement and another three in height and in which more than 250 people work. As the smart building is such a large building it is difficult to monitor and control the temperature of its interior throughout the year, we would like to make the case for this research in this building. To monitor and control the temperature in the smart building, a mesh was placed in the IoT devices of the smart building with the help of laser levels, the IoT devices were placed vertically—one in every section of the building. A total of 25 IoT nodes were deployed. The smart building where the case study was deployed is shown in Figure 4.

The type of sensor deployed in the building was a combination of the ESP8266 microcontroller in its commercial version “ESP-01” and a DHT22 temperature and humidity IoT node. The sum of both allows us for greater flexibility (e.g., by comparing the characteristics of this sensor with its previous model, the temperature measurement range of the DHT22 sensor is from 0${}^{\circ }$ to 50${}^{\circ }$ Celsius and the accuracy is ± 0.5${}^{\circ }$ Celsius) when collecting data and adaptability to the case study, since the DHT22 sensor is designed for indoor spaces (it has an operating range of 0 ${}^{\circ }$C to 50 ${}^{\circ }$C) according to its datasheet. The microcontroller obtains data from this sensor through the onewire protocol and communicates it to the network via Wi-Fi using HTTP standards and GET/POST requests. The ESP-IDF programming environment provided by the manufacturer of the microcontroller was used to program the device.

**Figure 5:**
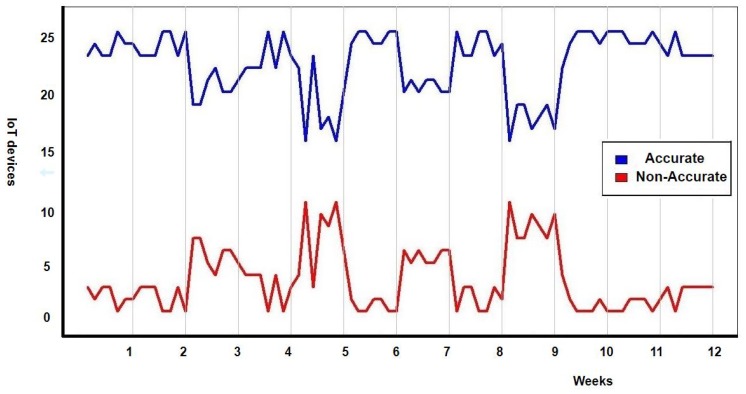
Number of accurate and inaccurate sensors during the 12 weeks of the case study.

During the design of this experiment, it was established that the data collection period would last three months in the smart building. It was also decided that the temperature would be measured and recorded in order to validate the accuracy of IoT temperature devices. It was decided during the design of the experiment that disturbances would be introduced into the system. These disturbances consisted of opening windows or randomly modifying the thermostats in the different zones of the smart building to induce these sensors to collect temperatures outside the range they were regularly collected (i.e., to simulate the inaccuracy of the sensors). It was proposed in the experimental design that disturbances would be introduced at weeks 3, 5, 7 and 9. To validate whether IoT devices were accurate or not, the consensus algorithm developed by Casado-Vara et al. was used [6]. This consensus algorithm compares the temperatures of the sensors with the temperatures of the nearest sensors that and using a consensus algorithm based on game theory decides which sensors are accurate and which are not. Once the data was collected from IoT devices for three months at one hour intervals, the consensus algorithm was applied to search for accurate and inaccurate sensors. The number of accurate and inaccurate sensors can be found in Figure 5. There are 25 sensors in this case study, so the number of accurate sensors is correlated with the number of inaccurate sensors since accurate sensors + inaccurate sensors = 25. Figure 5 illustrates the four random disturbances introduced in the IoT system. After each disturbance of the consensus algorithm, developed by Casado-Vara et al., detected that there were inaccurate sensors and maintenance staff were alerted to fix or replace them. Thus, it is possible to notice that, in weeks 3, 5, 7, and 9, the number of precise sensors decreases, but, a few days later, the number of precise sensors increases again; this is due to the external intervention of the maintenance staff in the system.

These two simulations have been done in the smart building described above. The aim of these two simulations is to validate the efficiency of the proposed model in 2 situations: Without human intervention and with intervention. In the case of simulation with intervention, the faulty sensors are repaired by the workers and become accurate sensors again. Whereas in the case study in which there is no human intervention, the system follows its own evolution. In the study of the populations of AD and NAD in IoT networks, we choose the following $g_{\beta }$ function:

(23) $$g_{\beta }\left(t\right)=\frac{1}{2e^{\beta \;t}\;-1}.$$

This function fulfills the conditions established for the variable $\beta_{g}$ and therefore will allow us to validate the model proposed in both cases (i.e., with human intervention and without intervention) and verify the results of the proposed theorems. In this LV system, varying the values of the variables a,b,c,d we have done two simulations to check that the theorems shown in the previous section are true.

### 3.2. Case Study Results

The results of the simulations that had been carried out using the data collected in the IoT network have a double purpose. The aim is to mathematically validate the LVNA model proposed in this paper and to study the behavior of IoT devices in an IoT network in order to predict their reliability over time. To demonstrate the efficiency of the LV model that we have designed for IoT networks in smart buildings, we are going to make two illustrative examples. The objective is to study the system behavior of Equation (3), which is the one in which trajectories do not remain in a $\Omega_{j}$ for a sufficiently long *t*, nor converge to a point of the set *A*. In fact, it could happen that trajectories would visit all $\Omega_{j}$ infinite times. This happens with β=1. In this case, the system Equation (3) is transformed into a conventional LV system. On the other hand, the system has oscillations in the predator and prey populations until they are buffered and equilibrium is reached for both prey and predator populations. This means that both species will find a point where they will be able to live together. In this way, the study of the behavior we have done in simulation 1, in which there is no human intervention, gives us the (expected) result that, in IoT simulation network 1, there is NAD and AD at the same time.

**Figure 6:**
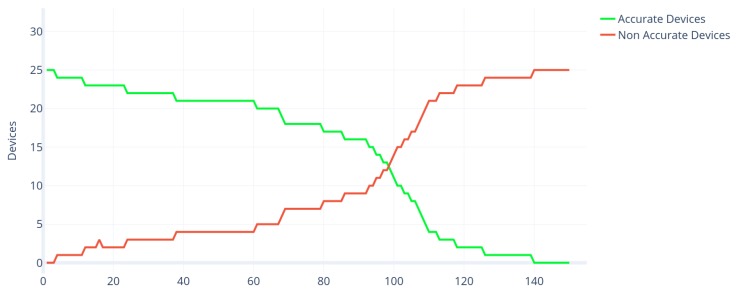
Time evolution of prey (accurate devices) and predator (non-accurate devices) absolute populations of simulation 1 of the Lotka Volterra Non Autonomous model proposed in this paper. The predator population (red line) grows as AD star to fail, reaching a point where no prey (AD) populates the system. In this simulation, human intervention is not taken into account; therefore, no NAD turns to AD after the stabilization in the IoT network

The results of the absolute population of the predators and prey obtained in simulation 1 are shown in Figure 6. The IoT network of simulation 1 is isolated (i.e., has no interaction with other IoT networks) and has no external human intervention. Therefore, the result that can be seen in the figure is that, with the passage of time, the predators (NAD) end up consuming all the prey (AD), and this makes the system reach equilibrium for both species when the predator has consumed all the prey.

**Figure 7:**
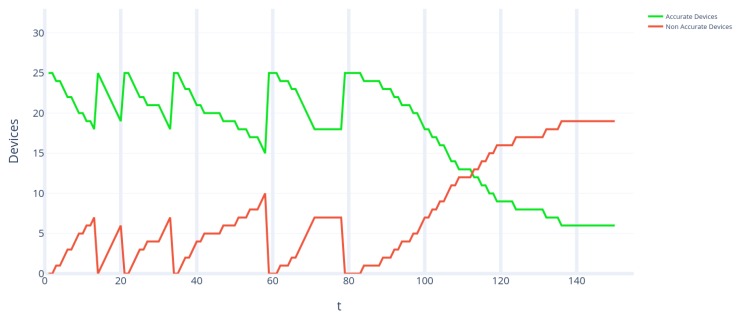
Time evolution of prey and predator populations of simulation 2 of the Lotka Volterra Non Autonomous model proposed in this paper. The predator population (red line) has fluctuations in its population until it reaches equilibrium while the prey population (green line) also has fluctuations in its population due to interaction with predators. In this simulation, human intervention is taken into account; therefore, when NADs are arranged the population of prey increases a lot (e.g., this can be seen in the peaks of both lines) while that of predators drops sharply.

In simulation 2, the same IoT network is presented, but, this time, there is human interference in the predator–prey population dynamics. Due to the more complex nature of this simulation, we will first discuss the population density obtained with the LV equations before commenting in Figure 7. On this occasion, u(t) suffers disturbances until its solution is stabilized at $u_{\infty }=7$, while v(t) has disturbances that vary between some values and periods in which they are 0. This is a clear example of the atto-fox problem, described in 2015 by Claude Lobry and Tewfik Sari [36], where the population density of a species reaches a minimum so small that it can’t modelize a real population. In this case, the “atto-sensor" problem is caused because the predator dynamics are involved in prey dynamics (i.e., the number of NAD + AD = 25 in this case study), destabilizing the system. Therefore, human intervention is required to repair the system, which can be seen clearly in the figure in the rapid growth of the prey (green curve) and in the sudden descents of the predator (red curve). In this way, human intervention stabilizes the system until a balance is reached between both populations of NAD and AD. Regarding the absolute population, we can observe the same fluctuactions produced by human intervention, but without reaching an asymptotic minimum. The disturbances are cushioned over time until they reach a state of equilibrium in AD = 6.

## 4. Conclusions

We extend the LV equations from community ecology to an ecosystem model based in an IoT network in a smart building to predict the system and the reliability of its IoT devices. The IoT network model comprises a set of differential equations concerned with the relationship between the IoT devices and the IoT network. We show the IoT model as an LVNA system in system evolution prediction through trajectories and equilibrium points analysis in $C_{1}$. To demonstrate the efficiency of the proposed model, we create two simulations to predict the reliability of the IoT network and its IoT devices. We associate every parameter in the LV IoT network model with its causal factors (i.e., accuracy and non-accuracy). The values of the parameters of the LV equations in the IoT ecosystem model help designers identify the key components of this system and make informed R&D investment and outsourcing network decisions. The mathematical analysis of the LV IoT ecosystem model offers designers’ guidelines on effective strategies to boost IoT network reliability in smart buildings.

Regarding the ecological analysis of the results, we can conclude that IoT sensors can be studied as biological populations. In Figure 6, the similarity between IoT sensors and predator–prey dynamics is demonstrated; this figure shows a growing amplitude oscillation that leads to the extinction of the predator; this type of response is typical to efficient and selective predators that drive the prey population below a capture threshold, happening when the prey has a density-dependent species and has a functional response [37]. Meanwhile, in Figure 7, the “atto-sensor” problem can be explained by the substitution of the malfunctioning hardware, driving the population to 0, and the emergence of new NAD once some time has passed.

Our paper has several limitations that offer opportunities for future research. Because of data limitations, we make simulations to demonstrate the application and effectivity of our IoT ecosystem model. We could apply this ecosystem model in emerging systems, such as smart cities, in the future. Such applications may derive compelling prediction results and help designers generate effective strategies to boost the reliability of smart cities.
